# *Sankofa*: Learning From the Past to Build the Future—Introduction to the Special Issue on Aging in Sub-Saharan Africa

**DOI:** 10.1093/geroni/igae031

**Published:** 2024-04-16

**Authors:** Jennifer Tehan Stanley, JohnBosco Chika Chukwuorji

**Affiliations:** Department of Psychology, University of Akron, Akron, Ohio, USA; College of Human Medicine, Michigan State University, Flint, Michigan, USA; Department of Psychology, University of Nigeria, Nsukka, Enugu, Nigeria

*Sankofa* is a concept derived from the Akan people of West Africa that highlights the importance of learning from the past to build the future. In Akan language, the African proverb expressing the concept is expressed as, “so wo were fi na wosan kofa a yenki,” which translates in the English language to, “it is not taboo to go back and fetch what you forgot.” In this special issue on Aging in Sub-Saharan Africa, we hope readers will learn about the past and present challenges and opportunities that older adults face in sub-Saharan Africa. We hope the varied stories, voices, and data compiled in this special issue will inspire researchers, policymakers, and thought leaders to build the future of aging in sub-Saharan Africa to align with the goals of the United Nations *Decade of Healthy Aging* (2021–2030) by improving the lives of older people, their families, and their communities.

Aging in sub-Saharan Africa is a relatively neglected area of research despite the increasing proportion of the population aging. As of 2020, more than 50 million people in the region are aged 60+ and this region has the most rapidly aging population in the world (He & Adjaye-Gbewonyo, 2020). Population aging is a policy concern throughout the world, but the challenges differ by region due to cultural, social, and historical differences. For example, recent recommendations on research with older persons living with HIV in Africa highlight the need for region-specific approaches to improving quality of life (Siedner, 2019). The notion that it is imperative to situate our understanding of development within a sociocultural context is at the core of the lifespan developmental perspective, which emphasizes the importance of considering how the historical and cultural context shapes development (Baltes, 1987). This collection of papers highlights how the varied cultures in sub-Saharan Africa shape development in later adulthood and emphasizes the critical need for research on aging to consider the sociocultural context. We hope by sharing this collection of papers on aging in sub-Saharan Africa we amplify the need for more research on the issues faced by the aging population in this region. Translational and innovative research on aging in sub-Saharan Africa is a critical need to inform clinical interventions and policies. By bringing awareness and interest to these topics, we hope to encourage continued quality research on aging in sub-Saharan Africa, more funding, and more strategic and collaborative research opportunities focused on these topics and populations.

The papers in this special issue are diverse in topic, format, population, authors, methods, and geography, reflecting the diversity of cultures in Africa with its many different languages, religions, histories of colonization, and educational practices (Chukwuorji et al., 2023). This special issue includes 18 papers with contributing authors from many countries including, Nigeria, Canada, Kenya, United States, and Hong Kong. Across these papers, participants lived in the following countries: Nigeria, Kenya, Ethiopia, South Africa, Ghana, Uganda, and Senegal. Many disciplines are also represented in this group of studies, including public health, psychology, social work, economics, and rehabilitation science. Importantly, several papers in this special issue address mental health topics, which were noted to be lacking in a recent review of research in sub-Saharan Africa (Saka et al., 2019). Other topics include public health, physical health, retirement, end-of-life care, caregiving, social support, age-friendly cities, and rural versus urban aging. Despite the diversity in topic and approach, four themes emerged among the papers:

1. The importance of place, religion, and culture.
2. Psychometrics and building research capacity.
3. Community engagement and social support.
4. Adapting to changing times.

We review each of these themes subsequently, highlighting the contributions of each paper as well as the complexity of the aging landscape in this region.

## The Importance of Place, Religion, and Culture

Ranging from investigations about rural versus urban differences among older Africans, to studies of religiosity and how it influences well-being, the importance of place, religion, and culture is exemplified by six of the papers in this special issue. For example, Burns and colleagues (Burns, 2024) explore how place and gender interact when examining disability prevalence among older adults in Ghana. By examining data with an intersectional lens, Burns and colleagues report that the disability disadvantage for urban women could be explained by marital status, where widowed urban women were more likely to experience disability than their married counterparts. However, marital status did not explain disability risk among rural older women. The authors conclude that these differences underscore the importance of taking an intersectional approach when considering the unique challenges of older adults experiencing disability. In another comparison of rural versus urban aging, Kaufman and colleagues (Kaufman, 2024) report important gender differences in social support exchanges among older adults living with HIV in Uganda and South Africa. Women are more involved than men in providing and receiving care in both countries, but the nature of the social support networks and care differ by setting, with greater interdependence in the more rural Ugandan sample than the more urban South African sample.

Rich qualitative data from five papers further elucidate the influence of culture on perceptions of illness, the dying process, caregiving, and coping. First, in a qualitative study examining how older chronically ill Nigerians cope with their diagnosis, Mahmoud and colleagues (Mahmoud, 2024) found that older Nigerians do not want individuals outside their close family circle to know that they are ill. Individuals conceal their illness because they fear discrimination and want to maintain normalcy when interacting with others. This study highlights the importance of considering the sociocultural context to understand the illness experience. Second, using a hermeneutic phenomenological research design, Ezulike and colleagues (Ezulike, 2024) explored the motives of older adults in Southeast Nigeria for providing informal care. Many aspects of African culture could contribute to older adults’ motives to provide care, including an African collectivist philosophy, as well as traditional African ethics where harmony is a virtue. Four themes emerged from older caregivers who were interviewed: reciprocity of kindness, altruism, sense of moral responsibility, and eagerness for peaceful longevity. Both religion and culture shaped these motivations. Unlike findings from Western cultures, informal caregiving is revered in African culture. Third, in a qualitative descriptive inquiry, Iwuagwu (2024) captures the voices of older grandmothers who serve as postpartum caregivers for their daughters, a practice called Ọmụgwọ. Three major themes emerged: positive outlook and continuity, cultural influences, and indirect care reward/benefits for grandmothers. Like other papers in this special issue, cultural reverence for the practice of Ọmụgwọ in Africa are important piece of the motivation for this continued practice of unpaid labor. Fourth, Silverstein and colleagues conducted semi-structured interviews with individuals involved in palliative care in Senegal to understand the barriers and opportunities for this important specialty of medical care in the region (Silverstein et al., 2024). Their findings resonate with other papers in this special issue, where culture and religion play an important role in shaping how palliative care is perceived. For example, what constitutes a good death varies across cultures. Palliative care is very new in Senegal and there is high demand for this type of care. Echoing other findings reported in this issue, some barriers include a cultural norm for medical secrecy and a hesitancy to talk about death.

In the fifth paper, Ukeachusim and colleagues (Ukeachusim, 2024) interviewed 71 widowed older adults in Southeast Nigeria and found the important role that spirituality and religious participation play in supporting the emotional well-being of widowed adults. Together, this group of papers amplifies the voices of older Africans, underlining the importance of considering aging in a cultural context.

## Psychometrics and Building Research Capacity

Four empirical articles on psychometrics and building research capacity for aging research and practice in Africa follow. In the first, Kalu et al. (2024) compared self-reported and capacity-based measures of mobility in community-dwelling older adults and explored age, cognitive status, and chronic conditions as mediators. The relationship between self-reported and capacity-based mobility outcomes in Nigerian older adults was lower than those in developed countries. This finding is considered important to the development of theories to further examine the complexity associated with mobility by laying the groundwork for investigating mobility variables that may serve as predictive mediators for mobility outcomes. The second article by Maina and colleagues (Maina, 2024) reported the development and validation of a measure of older people’s health and well-being in Kenya. The new tool demonstrated high internal consistency and convergent validity with the potential for utility in future research to inform policy and interventions. The 3^rd^ article by Akinrolie and colleagues (Akinrolie, 2024) was an ambitious work that reviewed existing studies on aging in Africa that used longitudinal data sets. An important suggestion by the authors emphasized the need to create funding competitions that concentrate on carrying out longitudinal analyses in Africa to clearly understand changes in the African aging population across time. In the same vein, Nagarajan and colleagues’ (Nagarajan, 2024) study describes the Longitudinal Study of Health and Ageing in Kenya. They highlighted the preliminary activities leading to the full population-representative study.

## Community Engagement and Social Support

One area of limited research on aging in Africa is community-based research and engagement of older adults. Three articles in our special issue provided interesting insights in this direction. In a sample of community-dwelling older adults, Olawa (2024) examines the connection between satisfaction with children’s achievements and health outcomes. They found that regardless of how much assistance their children are thought to have given, parents who are happy with their children’s accomplishments might feel good and, as a result, be in excellent health. Igbokwe and colleagues (Igbokwe, 2024) focused on how health behaviors and self-esteem explain the association between social support and successful aging. Their study which was conducted among Nigerian retirees highlighted the value of support from family and close friends in fostering a healthy lifestyle and boosting self-esteem as routes to aging successfully in Africa. Odeyemi et al.’s (2024) article advanced an innovative and highly promising approach known as the Citizen Science Project to foster collaborations between scientists and older adults in knowledge generation. Based on the findings of their study, it is critical for older adults to have access to both social and physical environmental elements that promote physical activity in their communities.

## Adapting to Changing Times

A final theme that emerged from the papers in this special issue is how individuals adapt to changing times. From individuals facing the transition to retirement to the impact of policy changes in pensions on the health and health behaviors of individuals, these papers show that older adults in sub-Saharan Africa are faced with dynamic issues and must adapt as they develop. For example, in a cross-sectional study of over 200 nurses in Nigeria, financial self-efficacy and pre-retirement planning were found to be important predictors of a positive perception towards retirement (Ujoatuonu et al., 2024). Beyond individual psychological factors that contribute to retirement outcomes, country-level policies can also influence this transition. Data from over 1,000 older men from a cohort study in South Africa examined some potential unintended consequences of recent expansions in pension eligibility on hypertension (Chang et al., 2024). The study found that while the expanded pension eligibility will likely have long-term positive effects, there was a small short-term negative effect on hypertension among older men. The authors speculate that greater income may lead to worse health behaviors such as the consumption of high-calorie foods among rural older men. In a similar examination of the effect of pension on health behaviors, Jock and colleagues investigate the frequency of alcohol use before and after pension receipt in a longitudinal population-based study of about 950 older men in South Africa (Jock et al., 2024). They report that employed men above the age of the Old Age Pension Grant eligibility of 60 years reported consuming almost five times more alcohol than employed men below the threshold. Clearly, as the proportion of the population of sub-Saharan Africa becomes increasingly older, individuals must adapt to the challenges of this changing landscape. One tool for improving adaptation in this dynamic environment is to strengthen research and education surrounding aging. Adamek and colleagues (Adamek, 2024) summarize the unique and common challenges that older adults face in Ethiopia and outline a positive path forward. They emphasize the importance of combating negative attitudes about older people, strengthening education and research, and developing policies and services for older adults. In this way, policies and education can support older adults in contributing to their communities.

## Conclusion and Outlook

Readers of this special issue will engage with innovative approaches, interesting and important findings, and critical next steps in research and policy development for this region. The four themes that emerged across the 18 papers in this special issue overlap. The first themes focused on the importance of place, religion, and culture. The next theme highlights the importance of strong psychometrics in our study of aging in sub-Saharan Africa and the need to build research capacity. These foundational papers help lay the groundwork for future research and collaborations to strengthen the research on aging in this region. The final two themes of community engagement and social support, and adapting to changing times, are both complex and necessary levels of analysis for a full understanding of the individuals and communities who are aging in sub-Saharan Africa. Taken together, all the papers in this issue contribute to what we know about how social and cultural factors shape development.

The next steps in this area should engage more researchers in studying context-specific factors in lifespan development. Future work would benefit from more team science, citizen science projects, funding, and an overall increase in collaboration. Following the concept of *sankofa*, researchers should incorporate what we have already learned about aging in sub-Saharan Africa to build the future of aging in this region to improve the lives of individuals and their communities.
