# Correction to: *Sankofa*: Learning From the Past to Build the Future—Introduction to the Special Issue on Aging in Sub-Saharan Africa

**DOI:** 10.1093/geroni/igae041

**Published:** 2024-04-26

In the published article titled, “*Sankofa*: Learning From the Past to Build the Future--Introduction to the Special Issue on Aging in Sub-Saharan Africa” *Innovation in Aging*, Volume 8, Issue 4, 2024, https://doi.org/10.1093/geroni/igae031, the coauthor J. C. Chukwuorji’s first affiliation had an incorrect city. The correct affiliation is as follows:

 “College of Human Medicine, Michigan State University, Flint, Michigan, USA”

